# Substantial out-of-pocket expenditure on maternity care practitioner consultations and treatments during pregnancy: estimates from a nationally-representative sample of pregnant women in Australia

**DOI:** 10.1186/s12884-017-1297-5

**Published:** 2017-04-12

**Authors:** Jon Adams, Amie Steel, Jane Frawley, Alex Broom, David Sibbritt

**Affiliations:** 1 Australian Research Centre in Complementary and Integrative Medicine (ARCCIM), Faculty of Health, University of Technology Sydney, Ultimo, NSW Australia; 2 Office of Research, Endeavour College of Natural Health, Level 2, 269 Wickham St, Fortitude Valley, QLD Australia 4006; 3 School of Social Sciences, The University of New South Wales, Sydney, Australia

**Keywords:** Pregnancy, Economics, Complementary and alternative medicine

## Abstract

**Background:**

A wide range of health care options are utilised by pregnant women in Australia. The out-of-pocket costs of maternity care in Australia vary depending on many factors including model of care utilised, health insurance coverage, and women’s decision to access health services outside of conventional maternity care provision.

**Methods:**

Women from the 1973–78 cohort of the Australian Longitudinal Study on Women’s Health (ALSWH) who identified as pregnant or as recently having given birth in 2009 were invited to complete a sub-study questionnaire investigating health service utilisation during their most recent pregnancy.

**Results:**

A total of 1,835 women agreed to participate in the sub-study. The majority of women (99.8%) consulted with a conventional health care practitioner during pregnancy, 49.4% consulted with a complementary and alternative medicine practitioner at least once during pregnancy and 89.6% of the women used a complementary and alternative medicine product. Women reported an average of AUD$781.10 in out-of-pocket expenses for consultations with conventional health care practitioners, AUD$185.40 in out-of-pocket expenses for consultations with complementary and alternative medicine practitioners and AUD$179.60 in out-of-pocket expenses for complementary and alternative medicine products. From the study data we estimate Australian pregnant women spend over AUD$337 M on out-of-pocket health services.

**Conclusion:**

While the majority of pregnant women in Australia may obtain health services via the publically-funded health care system and/or private health insurance coverage, our analysis identifies substantial out-of-pocket expenditure for health care by pregnant women – a trend in public spending for maternity care of importance to policy makers, health administrators, and health professionals.

## Background

Pregnant women in Australia have a wide range of maternity care practitioners and treatments available to them dependent upon access and affordability [1]. In 2009 nearly 294, 540 women gave birth in Australia [2]. The latest empirical data shows 90.1% of Australian women consult a general practitioner as part of their maternity care, 85.2% consult an obstetrician, 64.7% consult a midwife and 49.4% consult with a complementary and alternative medicine (CAM) practitioner [3]. Some of these consultations are provided via the publically-funded health care system (e.g. midwifery and general practice services), others attract private health insurance rebates and other services and treatments remain dependent upon direct out-of-pocket expenses.

Legislative changes have impacted on funding for health services in Australia in recent years, with a particular focus on incentivising the uptake of private health insurance by the general population [4]. Since implementation of these changes in 2005 [5] there has been a consistent rise in the number of privately insured Australians from less than one third of the population before the subsidies, to 51.6% in 2010 [6], and observed changes in maternity services [7]. Of those women accessing private health care services for maternity care, a health insurer covers 88%, whilst the remaining 12% incur out-of-pocket expenses [8]. Australians with private health insurance are also more likely to access CAM; however, most of these products and services remain a direct out-of-pocket expense for pregnant women [9]. In 2005, the per capita expenditure on CAM within the general Australian population was estimated at $182 on CAM products and $264 on CAM practitioners [9].

Although existing research has explored a range of pertinent issues around pregnancy in Australia [10–17], one area of health care utilisation during pregnancy not examined in any detail to date has been out-of-pocket expenditure. This is an important issue for maternity care practice and policy development and has implications for both pregnant women’s access to care and equity of care. We here draw upon data collected from a large, nationally representative sample of pregnant women to provide an estimation of the out-of-pocket expenses associated with the range of maternity health services available to pregnant women in Australia.

## Methods

The sample was derived from the Australian Longitudinal Study on Women’s Health (ALSWH) - a nationally-representative longitudinal population-based study examining the health of over 40,000 Australian women who were randomly selected from the Medicare database. The sub-study sample analysed in this paper was drawn from the ALSWH women born in 1973–78 who, in 2009, were identified as being pregnant or had recently given birth (*n* = 2,445). We invited this group of women to complete the sub-study survey in 2010, which examined a range of aspects associated with their health care during the pregnancy and birth of their youngest child. The study methodology has been described in full elsewhere [18]. The project has obtained ethical approval from University of Newcastle (#H-2010_0031), University of Queensland (#2010000411) and University of Technology Sydney (#2011-174 N), and all participants provided informed consent before taking part.

### Demographic characteristics

The women were asked about their highest educational qualification attained (post-graduate university degree; under-graduate university degree; certificate/diploma; trade/apprenticeship; higher school certificate; school certificate; no formal qualifications), current marital status (married/de facto; never married; divorced; separated; widowed) and income management (always difficult to manage on available income, sometimes difficult to manage on available income, managing on available income is not too bad, easy to manage on available income). They were also asked about their level of health insurance at the time of the pregnancy and birth of their youngest child (yes, full coverage including pregnancy-related care; yes, not including pregnancy-related care; no), number of previous births, and area of residence (urban or rural). Postcode of residence was used to classify area of residence as urban or non-urban.

### Health service use and expenditure

Women were asked to indicate the frequency of their consultations with conventional health care practitioners (i.e. general practitioner, obstetrician, and midwife) and CAM practitioners (e.g. acupuncturist, chiropractor, and naturopath) for pregnancy-related health conditions. The women were also asked to indicate if they had used a range of CAM products (e.g. herbal medicine, vitamins/minerals, and aromatherapy oils) for pregnancy related conditions.

Out-of-pocket expenditure was reported as a categorical variable. Median values were also attributed to each category to enable extrapolation of expenditure. The categories are: *less than $50* (median: $50); *$100-$499* (median: $300); *$500-$999* (median: $750); *$1000-$1499* (median: $1250); *$1500 or above* (median $1500).

### Statistical analyses

Frequencies and percentages were used to report on the rates of health service utilisation. Expenditure was estimated based upon midpoints of women’s reported out-of-pocket expenses. Extrapolation of these expenses for the Australian population was based upon the number of women who gave birth in Australia in 2009 [2].

Women were grouped in to categories based on their reported level of expenditure (none = $0; low = $50; medium = $300-$750; high = $1250-$1500). The relationship between demographic characteristics and level of expenditure was determined through logistic regression.

## Results

A response rate of 79.2% (*n* = 1,835) was attained. The majority of the women (99.8%) consulted with a conventional health care practitioner and almost half of the women (49.4%) consulted with a CAM practitioner at least once during pregnancy for pregnancy-related issues. In addition, 89.6% of the women used CAM products during pregnancy for pregnancy-related issues. The demographic characteristics of the women are presented in Table 1.

**Table 1:** Demographics of participants

Demographics	All participants (*n* = 1,835)^a^	
	n	%
Area of residence		
Urban	1134	62.4
Rural	629	34.6
Remote	55	3.0
Number of children		
None	89	4.9
One	697	38.0
Two	700	38.2
Three or more	349	19.0
Marital status		
Married/De facto	1760	96.3
Separated/Divorced/Widowed	46	2.5
Never married	21	1.2
Qualifications		
Year 12 qualification or less	292	16.0
Apprenticeship or Diploma qualification	435	23.9
Undergraduate/postgraduate university degree	1095	60.1
Private health insurance with maternity/birth cover		
Yes	1139	62.5
No	684	37.5
Conventional maternity health professionals		
General practitioner	1562	90.1
Obstetrician	1416	85.2
Midwife	983	64.7
Birth environment		
Private hospital (or private patient at public hospital)	850	46.9
Public hospital	882	48.7
Birth centre/community	80	4.4
	mean	SD
Age	34.95	2.30

^a^Not all participnts answered all questions

The women incurred an average of AUD$781.10 in out-of-pocket expenses for consultations with conventional health care practitioners, AUD$185.40 in out-of-pocket expenses for consultations with CAM practitioners and AUD$179.60 in out-of-pocket expenses for CAM products. Extrapolation of these out-of-pocket expenses to the population of women who gave birth in Australia in 2009 (*N* = 294,540) [2] provides the following estimates: AUD$230,065,194 in out-of-pocket expenses for consultations with conventional health care practitioners; AUD$54,607,716 in out-of-pocket expenses for consultations with CAM practitioners; and AUD$52,899,384 in out-of-pocket expenses for CAM products. That is, pregnant women in Australia incurred a total of $337,572,294 in out-of-pocket expenses for pregnancy-related health care of which $284,672,910 (84%) was spent on consultations with a health professional and $107,507,100 (32%) was spent on some form of CAM.

Table 2 reports the relationships between women’s level of expenditure and their demographic characteristics. Women who had a medium level of expenditure on CAM practitioners were more likely to have ancillary private health insurance coverage (AOR 2.46; CI 1.23-4.93, *p* = .011) but no other demographic characteristic was associated with CAM practitioner level of spending. Women who spent a small amount of money on CAM products were less likely to give birth in a birth centre or in the community (AOR 0.34; CI 0.15-0.76, *p* = .009), be working full time at the time of birth (AOR 0.63; CI 0.43–0.92; *p* = 0.022), or had consulted with a GP for pregnancy-related health conditions (AOR 0.80; CI 0.69–0.94; *p* = .007). Women who reported a medium level of CAM product expenditure for their maternity care were more likely to give birth at home or community (AOR 3.00; CI 1.38–6.53; *p* = .006), work full time (AOR 1.48; CI 1.00–2.18; *p* = .048), or to have seen either a GP (AOR 1.31; CI 1.12–1.53; *p* = .001) or an obstetrician (AOR 1.17; CI 1.01–1.36; *p* = .033), but were less likely to live in a rural environment (AOR 0.67; CI 0.47–0.94; *p* = .022). Those women who had a high level of self-reported spending on CAM products for pregnancy health were more likely to have seen a GP for their maternity care (AOR 2.31; CI 1.02–5.21; *p* = .042).

**Table 2:** Relationship between demographic characteristics and maternity health service expenditure (*n* = 1835)

Characteristic	Health service expenditure								
	Maternity care provider			CAM practitioner			CAM products		
	Low	Medium	High	Low	Medium	High	Low	Medium	High
	AOR (95% CI)	AOR (95% CI)	AOR (95% CI)	AOR (95% CI)	AOR (95% CI)	AOR (95% CI)	AOR (95% CI)	AOR (95% CI)	AOR (95% CI)
Education									
Up to year 12 or equivalent	ref	ref	ref	ref	ref	ref	ref	ref	ref
Trade/apprenticeship/certicipate/diploma	0.53 (0.21–1.34)	1.13 (0.62–2.06)	1.09 (0.55–2.19)	1.17 (0.55–2.55)	1.16 (0.63–2.12)	0.64 (0.04–10.94)	1.59 (0.92–2.73)	0.66 (0.39–1.14)	0.61 (0.03–11.91)
University degree	0.53 (0.24–1.20)	0.97 (0.57–1.67)	1.33 (0.72–2.43)	0.68 (0.34–1.34)	1.40 (0.83–2.37)	2.11 (0.24–18.31)	1.02 (0.63–1.64)	1.13 (0.71–1.82)	0.53 (0.04–6.43)
Income manageability									
Impossible/very difficult to manage	ref	ref	ref	ref	ref	ref	ref	ref	ref
Difficult some of the time	1.31 (0.48–3.60)	0.65 (0.35–1.19)	1.32 (0.66–2.68)	0.55 (0.25–1.21)	1.14 (0.61–2.13)	0.37 (0.07–1.99)	1.03 (0.59–1.79)	1.10 (0.64–1.90)	0.45 (0.03–6.46)
Not too bad	0.97 (0.36–2.61)	0.78 (0.43–1.40)	1.16 (0.59–2.27)	0.65 (0.31–1.34)	1.29 (0.71–2.35)	0.27 (0.16–1.35)	1.36 (0.80–2.31)	0.91 (0.54–1.54)	0.39 (0.03–5.14)
Easy to manage	1.31 (0.42–4.06)	0.79 (0.40–1.53)	1.00 (0.48–2.12)	0.64 (0.27–1.51)	1.56 (0.79–3.05)	0.21 (0.03–1.56)	0.88 (0.48–1.60)	1.31 (0.73–2.37)	0.40 (0.02–7.97)
Private health insurance	0.83 (0.46–1.50)*	0.68 (0.42–1.10)	3.60 (1.76–7.35)*	0.58 (0.29–1.14)	1.45 (0.85–2.48)	1.38 (0.30–6.29)	1.22 (0.77–1.94)	1.04 (0.65–1.66)	0.25 (0.01–12.43)
Health insurance cover for ancillary services	0.65 (0.26–1.67)	0.95 (0.52–1.74)	1.47 (0.76–2.88)	0.62 (0.28–0.40)	2.47 (1.24–4.93)*	1.16 (0.13–10.11)	0.75 (0.43–0.94)	1.49 (0.86–2.60)	0.35 (0.03–3.72)
Health care card	0.40 (0.13–1.22)	1.24 (0.54–2.84)	1.65 (0.63–4.36)	2.02 (0.53–7.65)	0.44 (0.49–1.04)	–	1.57 (0.71–3.47)	0.60 (0.28–1.30)	–
Marital status									
Never married	ref	ref	ref	ref	ref	ref	ref	ref	ref
Married/defacto	0.81 (0.01–62.26)	0.73 (0.62–8.61)	2.29 (0.19–28.04)	0.40 (0.04–4.22)	2.30 (0.22–23.8)	–	0.70 (0.09–5.49)	0.91 (0.11–7.23)	–
Seperated/divorced/widowed	2.91 (0.03–285.81)	0.26 (0.02–4.12)*	3.69 (0.29–2.85)	0.45 (0.03–7.82)	1.74 (0.12–24.9)	–	0.64 (0.06–6.68)	1.06 (0.10–11.02)	–
Locality									
Urban	ref	ref	ref	ref	ref	ref	ref	ref	ref
Rural	0.36 (0.18–0.71)*	1.78 (1.22–2.57)*	1.02 (0.66–1.60)	1.21 (0.63–2.55)	0.83 (0.56–1.23)	0.40 (0.08–2.01)	1.36 (0.96–1.93)	0.67 (0.47–0.94)*	1.23 (0.17–8.81)
Remote	0.17 (0.03–1.09)	2.26 (0.89–5.83)	0.90 (0.29–2.85)	0.41 (0.08–1.95)	1.09 (0.42–2.84)	–	0.50 (0.19–1.26)	1.92 (0.79–4.64)	–
Birth environment									
Public hospital	ref	ref	ref	ref	ref	ref	ref	ref	ref
Private hospital	0.18 (0.08–0.43)*	0.31 (0.20–0.48)*	5.10 (3.21–8.09)*	1.26 (0.63–2.55)	0.90 (0.56–1.46)	1.86 (0.22–15.46)	0.68 (0.45–1.04)	1.27 (0.83–1.94)	0.15 (0.00–23.29)
Birth centre/community	0.46 (0.19–1.11)	0.53 (0.25–1.13)	10.46 (3.62–30.20)*	0.57 (0.17–1.87)	2.17 (0.91–5.17)	4.29 (0.75–24.66)	0.34 (0.15–0.77)*	3.00 (1.38–6.53)*	–
Work status before pregnancy									
Not working	ref	ref	ref	ref	ref	ref	ref	ref	ref
Full time work	1.81 (0.87–3.77)	0.71 (0.45–1.10)	1.17 (0.71–1.94)	1.70 (0.91–3.18)	1.30 (0.84–2.02)	1.06 (0.21–5.36)	0.63 (0.43–0.94)*	1.48 (1.00–2.18)*	0.45 (0.04–4.56)
Part time work	1.68 (0.81–3.51)	0.78 (0.37–1.69)	1.32 (0.56–3.12)	1.16 (0.60–2.25)	1.26 (0.81–1.97)	1.95 (0.46–8.25)	0.86 (0.58–1.28)	0.98 (0.66–1.45)	0.59 (0.06–5.63)
Casual work	1.25 (0.32–4.84)	0.79 (0.37–1.69)	1.32 (0.56–3.12)	1.51 (0.53–4.33)	1.27 (0.58–2.82)	–	0.93 (0.47–1.86)	0.88 (0.44–1.75)	–
Consultations with a maternity care provider									
General practitioner	0.60 (0.46–0.78)*	1.42 (1.20–1.68)*	0.89 (0.72–2.88)	0.97 (0.77–1.22)	1.12 (0.95–1.33)	0.96 (0.53–1.73)	0.80 (0.69–0.94)*	1.31 (1.12–1.53)*	2.32 (1.03–5.21)*
Obstetrician	0.60 (0.47–0.75)*	0.80 (0.69–0.94)*	2.18 (1.77–2.67)*	0.82 (0.66–1.02)	1.10 (0.93–1.29)	0.71 (0.38–1.32)	0.88 (0.76–1.02)	1.17 (1.01–1.36)*	2.50 (0.58–10.69)
Midwife	1.32 (1.08–1.62)*	0.89 (0.78–1.02)	0.92 (0.56–3.12)	1.08 (0.91–1.29)	1.05 (0.92–1.33)	1.16 (0.74–1.82)	0.97 (0.84–1.06)	1.11 (0.99–1.25)	0.29 (0.00–5.20)

*p = <0.05

Table 2 also reports the relationships between women’s level of conventional medial expenditure and their demographic characteristics. Women who had a low level of conventional medical expenditure were more likely to visit a midwife (AOR 1.33; CI 1.08–1.62; *p* = .006), and less likely to live in a rural setting (AOR 0.36; CI 0.18–0.71; *p* = .003), give birth in a private hospital (AOR 0.83; CI 0.46–1.50; *p* < .001), and visit a GP (AOR 0.59; CI 0.46–0.78; *p* < .001) or an obstetrician (AOR 0.59; CI 0.47–0.75; *p* < .001). Women who spent a medium amount of money on conventional services were also more likely to live in a rural area (AOR 1.77; CI 1.22–2.57; *p* = .003) and more likely to visit a GP (AOR 1.42; CI 1.20–1.68; *p* < .001). They were less likely to give birth in a private hospital (AOR 0.31; CI 0.20–0.48; *p* < .001) and/or visited an obstetrician (AOR 0.80; CI 0.69–0.94; *p* = .006). Women who had a high level of self-reported spending on conventional services were more likely to have seen an obstetrician (AOR 2.18; CI 1.77–2.67; *p* < .001), have private health insurance (AOR 3.60; CI 1.76–7.35; *p* < .001), give birth in a private hospital (AOR 5.10; CI 3.21-8.09; *p* < .001) or in a birth centre or the community (AOR 10.46; CI 3.62–30.20; *p* < .001).

## Discussion

This study highlights a substantive annual out-of-pocket expenditure by Australian women for pregnancy-related health treatments and services. This finding may be related to high rates of private obstetrics services being accessed by women [7], which may be linked to increased public uptake of private health cover [6] in line with the health insurance legislative changes in recent years [19]. Our findings confirm that women with private health insurance have much greater pregnancy-related out-of-pocket expenses from consultations with a conventional maternity care provider. Likewise, women who birth in a private hospital or the community are also more likely to have high out-of-pocket maternity care expenses. These findings create an interesting juxtaposition in that the obstetrician-led model of care women would have access to in a private hospital is vastly different to the midwife-led continuity of care model most common in home births [1, 20, 21].

There are also links in the findings between women’s out-of-pocket maternity care expenses and their maternity care provider which adds complexity to the interpretation: women are more likely to have high out-of-pocket expenses if they consult with an obstetrician but to have low out-of-pocket expenses if they consult a midwife. The seeming conflict between the findings associated with birth setting and maternity care providers may be explained by the models of care supported by government health funding in Australia [1]. The most common maternity care services accessed by Australian women are offered through public hospitals where, alongside obstetricians, midwives play a fundamental role in the service delivery [2]. In some circumstances, these services include midwife-led continuity of care models offered through a birth centre within the hospital [2]. These services are all fully government funded and result in no out-of-pocket expenses for women. In contrast, women accessing obstetrician-led maternity care in a private hospital will be commonly accessing private health insurance to reduce their costs; but while a health insurer covers 88% of their obstetric costs, 12% are out-of-pocket expenses [8]. In this context, it is important to note that the figure identified in our study does not include insurance premiums outlaid by women and as such our calculations may underestimate the true out-of-pocket expenses which individuals contribute to their maternity care. Meanwhile, there is a growing body of evidence of safety and effectiveness and a lower overall cost of care per birth associated with the midwife-led care for pregnant women at all levels of risk [17]. Despite this, it is worth noting that women who give birth in the community have a much greater likelihood of having high out-of-pocket expenses compared to women birthing in a private hospital. This finding is best explained by the low level of government funding for home birth programs in Australia [22] and inadequate provision for private health insurance rebates for women who birth at home.

Another important finding from our analysis is the significant out-of-pocket expense women are committing for CAM treatments and practitioners. Previous work estimates CAM expenditure in the Australian general population as AUD$4.13 billion [9] with mid-age women spending AUD$349 annually per capita on self-prescribed CAM [23]. Recent research has identified high CAM use by Australian women for pregnancy-related health conditions [3, 24] including the high rate of self-prescription of herbal medicine [12]. Our data provides the first differential breakdown of expenditure on both CAM products and practitioners and as such adds context to our understanding of CAM use in Australia and its perceived importance and value amongst the Australian population.

It is also interesting to observe that our study found a higher degree of spending on CAM products by women who were more likely to consult with a GP or obstetrician for their maternity care. Previous research has identified antipathy toward CAM by medical doctors [25]. As such, the relationship between greater spending on CAM by women consulting with a physician for pregnancy-related health is unexpected and is possibly driven by women rather than under the guidance of the practitioner. Given the reportedly low rates of disclosure of the use of CAM to obstetricians [26], this finding emphasises the need for medical doctors to inquire about CAM use when providing care to pregnant women.

The findings from this analysis may be affected by recall bias as the survey was prospective and self-administered. Previous maternity care research has examined the effects of recall bias and has identified that the more general aspects of health and care provision are less affected than recall of pregnancy-related health conditions [27, 28]. As such, recall bias for this analysis is not expected to be substantive. Furthermore, as any errors generated by the recall bias would occur equally across all subcategories of the study population the effect would be considered non-differential and the reported odds ratios are likely to be an under-estimate if they deviate from the true effect [29]. In addition, not all women who were eligible to complete the survey chose to do so and as such may expose the study to risk of sampling bias, whereby the sample is not representative of the whole population [29]. Fortunately, the size of this study and previous verification of the ALSWH study as nationally representative [30] offsets the potential impact of any actual sampling bias. Another possible limitation of this analysis may be linked to the age restrictions of the cohort which may have resulted in selection bias. While the average age of birthing women in Australia in 2009 was 30.0 years [2], which is within the range of women included in the study, it is acknowledged that not all Australian women who give birth are represented in the age group of the cohort. In light of these limitations, the expenditure figures presented should be viewed as estimates only. A comprehensive economic evaluation of Australian maternity care, which includes all elements of pregnancy-related health spending is needed, and this data provides insights which can inform such work.

## Conclusion

This article reports the first estimates of out-of-pocket expenditure drawing upon a large, nationally-representative sample of pregnant women. While the majority of pregnant women in Australia may obtain health services via the publically-funded health care system and/or private health insurance coverage, our analysis identifies substantial out-of-pocket expenditure for health care by pregnant women – a trend in public spending for maternity care of importance to policy makers, health administrators, and health professionals.

## Abbreviations

ALSWH: Australian Longitudinal Study on Women’s Health
CAM: Complementary and alternative medicine
